# Some theoretical insights into the hologenome theory of evolution and the role of microbes in speciation

**DOI:** 10.1007/s12064-018-0268-3

**Published:** 2018-07-31

**Authors:** Adrian Stencel, Dominika M. Wloch-Salamon

**Affiliations:** 1 Faculty of Philosophy, Jagiellonian University, ul. Gołębia 24, 31-007 Kraków, Poland; 2 Institute of Environmental Sciences, Jagiellonian University, Gronostajowa 7, 30-387 Kraków, Poland

**Keywords:** Holobiont, Symbiosis, Evolution, Hologenome

## Abstract

Research on symbiotic communities (microbiomes) of multicellular organisms seems to be changing our understanding of how species of plants and animals have evolved over millions of years. The quintessence of these discoveries is the emergence of the hologenome theory of evolution, founded on the concept that a holobiont (a host along with all of its associated symbiotic microorganisms) acts a single unit of selection in the process of evolution. Although the hologenome theory has become very popular among certain scientific circles, its principles are still being debated. In this paper, we argue, firstly, that only a very small number of symbiotic microorganisms are sufficiently integrated into multicellular organisms to act in concert with them as units of selection, thus rendering claims that holobionts are units of selection invalid. Secondly, even though holobionts are not units of selection, they can still constitute genuine units from an evolutionary perspective, provided we accept certain constraints: mainly, they should be considered units of co-operation. Thirdly, we propose a reconciliation of the role of symbiotic microorganisms with the theory of speciation through the use of a developed framework. Mainly, we will argue that, in order to understand the role of microorganisms in the speciation of multicellular organisms, it is not necessary to consider holobionts units of selection; it is sufficient to consider them units of co-operation.

## Introduction

In the 1960s, the scientific community was astonished by demonstrations of previously hidden genetic diversity, both within and between individuals. This diversity was uncovered by advances in experimental techniques such as gel electrophoresis, which revealed protein polymorphisms and DNA diversity, opening the way for empirical and theoretical research which led in turn to a more sophisticated understanding of how evolution takes place. At present, the scientific community is undergoing a similar experience. Current achievements in genomics and technology have led to surprising new discoveries that biologists may not have even dreamed of just a few decades ago (Koonin and Wolf 2012; Rose and Oakley 2007). Because of its high level of general interest and applicability, along with the ubiquity and relative accessibility of biological material, many of the recent discoveries in genomics have been made in the field of microbial ecology, particularly in the exploration of the relationships between hosts and their microbes. These discoveries have shown that populations and species of multicellular organisms such as plants or animals—which, following O’Malley and Dupre (2007), we will call *macrobes*—are much more genetically diverse than had been predicted based solely on comparisons of nuclear DNA sequences. This newly discovered heterogeneity is based on microorganisms or other units carrying information in the form of DNA such as plasmids or viruses (Hosokawa et al. 2006; Ley et al. 2006; Oh et al. 2010; Yatsunenko et al. 2012; Gilbert et al. 2012; Godoy-Vitorino et al. 2012; Linnenbrink et al. 2013). Furthermore, these microorganisms play many important roles in the biology of multicellular organisms; for example, they support the latter’s digestive processes (Ley et al. 2006; Linnenbrink et al. 2013), or they are needed for the proper development of the latter’s immune systems (Mazmanian et al. 2005).

Notwithstanding, symbiotic microorganisms are undoubtedly changing our understanding of the physiological properties of multicellular organisms. Another important issue is whether research on microbiomes is also changing our understanding of the evolution of macrobes. Here, the fundamental question is whether a host and its symbiotic microbes should be considered a single cohesive unit from the evolutionary perspective (see Suárez 2018, for a comprehensive review of this issue). One important step toward resolving this question was the formation of the hologenome theory of evolution (HTE) (Zilber-Rosenberg and Rosenberg 2008; Brucker and Bordenstein 2012, 2013). This theory states that a host is inseparable from its associated microbiome and that together they act as a ‘unit of selection’ (collectively, a *holobiont*) in evolution. Thus, the genome of the host and the genome of the microbiome (collectively, the *hologenome*) constitute the genetic basis for its evolution. This is an important addition to the theory of evolution, bringing symbiotic microorganisms into the picture and initiating a debate about their significance and role in the evolution of multicellular organisms, thereby enabling a holistic view of the nature of species—one that is increasingly appreciated by both philosophers and biologists (Ley et al. 2006; Gilbert et al. 2012; Pradeu 2010, 2011; Hutter et al. 2015), thanks to its basic emphasis on the fact that thinking of plants and animals, including humans, as autonomous biological individuals is a serious oversimplification. Furthermore, HTE has inspired many researchers to take microbes into account when conducting experiments, especially in the field of speciation (Brucker and Bordenstein 2012, 2013; Wang et al. 2015). Therefore, HTE is without doubt a reasonable research programme indicating new study directions and influencing the way research itself is conducted.

However, recently some scholars have argued that the hologenome theory is based on certain assumptions that may turn out to be wrong (Moran and Sloan 2015; Douglas and Werren 2016; Skillings 2016). Mainly, they question whether it is proper to assume that a host and its all symbiotic microorganisms constitute a unit of selection. Obviously there are examples of interactions characterised by such a high level of interdependence and connectedness that it is reasonable to consider the participants as a single unit that can be selected by natural selection as a whole (see the next section). However, other interactions between microorganisms and hosts are not as ‘tight’: microorganisms are not as important to the host, they can move from one host to another, etc. This leads some scholars to maintain that they are more accurately considered independent agents engaged in certain kinds of ecological interactions with their hosts (Moran and Sloan 2015; Douglas and Werren 2016). This situation is quite interesting, since, even though HTE seems to justify certain research aims, e.g. the above-mentioned studies on speciation (Brucker and Bordenstein 2012, 2013; Wang et al. 2015), there is some disagreement about the legitimacy of its basic theoretical assumptions. This may sound counterintuitive. After all, if something works, how can it be wrong? However, the history of science is full of theories that were useful for conducting scientific research for decades, but were later shown to be based on invalid assumptions (for a meta-analysis of such cases, see Laudan 1981). This observation from the history of science should therefore inspire us to analyse the assumptions of HTE in order to understand whether they should be retained, revised, or rejected.

Our aim in this paper is to follow the path of those scholars aiming to undermine the hologenome theory of evolution, since we believe that this theory’s basic assumption is not true. Mainly, we believe that it is very unlikely that a holobiont (defined, let us recall, as a host and all its symbiotic microbes) can act as a unit of selection. This concern has been raised recently by many scholars (Moran and Sloan 2015; Douglas and Werren 2016; Skillings 2016), raising an obvious question: what is to be done with the idea of the holobiont? Some interesting ideas have been put forward, suggesting that even though holobionts are not units of selection, they still can be considered genuine units from the perspective of other fields, such as immunity (see for example Pradeu 2011, 2016). This is very likely true; however, we are interested here in understanding the nature of holobionts from the evolutionary point of view. Thus, the question is: if it is true that holobionts are not units of selection, do they have any evolutionary meaning? We will argue that even though holobionts are not units of selection, they still can be considered important units from the evolutionary point of view. Mainly, they should be considered units of co-operation. The structure of this paper is as follows. In the next section, by referring to the recent work on natural selection by Godfrey-Smith (2009), we will show that it is very unlikely that a holobiont can act as a unit of selection. Then, we will argue that holobionts are still important from the evolutionary point of view. Mainly, we will argue, referring to the recent work of Queller and Strasmann (2009, 2010, 2016), that they constitute units of co-operation. Then we will argue that, in order to incorporate microorganisms into the theory of speciation, it is sufficient to consider holobionts units of co-operation. Having chosen the field of speciation deliberately, as the hologenome theory of evolution is currently being applied therein (Brucker and Bordenstein 2012, 2013; Wang et al. 2015), we will show that a refined concept of the holobiont can still be used to understand the role of microbes in the process of speciation.

## Can a holobiont act as a unit of selection?

The fundamental assumption behind the hologenome theory of evolution is that holobionts are units of selection (Zilber-Rosenberg and Rosenberg 2008; Brucker and Bordenstein 2012, 2013). Of course, as proponents of this theory argue, holobionts are not the only units of selection, and thus the fundamental assumption should be interpreted in the context of a multi-level theory of selection. Selection can occur simultaneously at the levels of the holobiont and of, for example, the host. This pluralistic approach toward holobionts can be seen in a paper by Theis et al. (2016, p. 4): ‘This strict claim leads biologists into error, as all of the literature emphasises that multiple levels of selection can operate simultaneously. For example, selfish genetic elements can be selected within a genome that is in turn selected for any number of phenotypes that affect fitness—this is uncontroversial. While the holobiont is posited to be “a unit of selection in evolution” (…) it is naturally not proposed as the only or necessarily primary unit of selection’

Unfortunately, the debate over units of selection is full of disagreements and philosophical issues (see Okasha 2006; Godfrey-Smith 2009). One popular approach aimed at understanding what a unit of selection is, championed by Dawkins (1976, 1982) and Hull (1980), distinguishes replicators and interactors. However, this approach was recently questioned by Godfrey-Smith (2009), who argued that it sometimes requires superfluous elements and who went on to propose a more general approach, the concept of *Darwinian individuals*. In this paper, we will follow the elaboration developed by Godfrey-Smith (2009), as it is the most detailed elaboration of the process of natural selection and thus will help us to understand whether or not a given holobiont is a unit of selection.

Darwinian individuals are entities capable of undergoing the process of natural selection; as such, they must be characterised by at least three properties: variation, differential reproduction (fitness differences), and heredity (Lewontin 1970; Godfrey-Smith 2009, 2012). Variation means that entities in a population vary in terms of their traits: height, rate of reproduction, biochemical pathways, etc. This variation in a population must contribute to differences in the fitness of those entities; this is the meaning of the next parameter, differential reproduction. Furthermore, in order for change to occur across generations, there must be a kind of heredity in the parent–offspring line, whereby parents are capable of passing on their traits to their offspring. If we combine these three minimal properties, we arrive at the conclusion that Darwinian individuals must be *reproducers*, i.e. units capable of reproducing (Griesemer 2001; Godfrey-Smith 2009; Stencel 2016), which are thus able to pass on their traits, via the process of reproduction, to their offspring. Thus, we can expect that a population of reproducers will evolve via natural selection, as the most successful reproducers will produce more offspring (which will inherit these traits) than others; accordingly, the relative frequency of traits will change over generations.

Darwinian individuals are not, of course, homogenous. Rather, they are capable of extreme variation between different lineages as a result of their constant evolution. To illustrate this, let us use a classification developed by Godfrey-Smith (2009), who distinguished three kinds of reproducers. The first are *scaffolded reproducers*: those entirely dependent on external machinery. This category includes viruses, as they need the biochemical machinery of other reproducers in order to reproduce. The next category is *simple reproducers*, which possess inner machinery and thus need only external resources to initiate reproduction. One example is a bacterial cell that needs nutrients in order to reproduce, but can dispense with the presence of other reproducers. The last category constitutes *collective reproducers*—in essence, entities that can reproduce themselves, but which are built of elements which can also reproduce themselves. One example is a multicellular being, such as a cat, which produces more cats by reproducing at the level of the cat, but which can produce as well more nervous cells by reproducing at the level of those cells.

From the perspective of this paper, the most interesting category is collective reproducers, because holobionts are composed of elements (microbes and host) which, as is well-known, can reproduce themselves. Thus, the question is whether holobionts are so well integrated that they can also reproduce at the level of holobionts. Are they collective reproducers, which could thus be considered units of selection? Before we answer this question, let’s take a closer look at the concept of a collective reproducer.

As stated above, a collective reproducer is a unit made of elements that can reproduce themselves, but is as well a unique aggregation of units with the ability to reproduce as a whole. Our body is a good example. Our cells can reproduce themselves; for example, muscle cells produce more muscle cells; but our body can also give rise to another body. Thus, we reproduce at the level of cells and at the level of human beings. The question is: why it is possible to reproduce at the collective level? Or, in other words: why are some collectives able to reproduce at the level of the whole, such as a single human being, while other collectives, such as a group of human beings inhabiting a given city, cannot reproduce at the level of a community? Some units can reproduce as units because they are made of elements that work together in order to produce more such units. This is not, of course, because these elements ʻwant’ to do so voluntarily, but because they have ‘got stuck’—due to their evolutionary history—in the same body. In other words, these units cannot survive and reproduce on their own because their capacity to enjoy a free-living lifestyle has been lost in the process of evolution, which favoured integration over generations.

Therefore, a collective reproducer is an evolved entity, a group of elements that are, as a result of their common history, very tightly integrated, to the extent that they are oriented toward enhancing the reproduction of the collective. Of course, before a group of elements can achieve a high level of integration, they must lose many traits that may be harmful from the perspective of the whole, but they must also evolve many traits to make the whole cohesive. This does not have to be a rapid process, but it may take some time for two units to achieve so high a level of interdependence that they work as a single reproducer. Thus, in nature, units may exist at different stages in transition, ranging from simple to collective reproducers.

Now the question is: are holobionts at the very last stage of this process? In other words, are they so tightly integrated that they should be seen as representing a new level at which collective reproduction occurs? If we look at the relationships between hosts and symbiotic microorganisms, we realise that, indeed, there are many examples of interactions that have developed a level of interdependence leading to the emergence of collective reproducers. For instance, eukaryotic cells originated as collective ‘multi-species’ reproducers, because they resulted from the symbiosis of a host cell and engulfed bacteria (Margulis 1993). Currently, these bacteria constitute cell mitochondria, which form the foundations of the biochemical machinery of eukaryotes. However, these cellular structures are so well integrated into the structure of eukaryotic cells that it is hard to believe that their ancestors enjoyed a free-living lifestyle. This is the consequence of the fact that these bacteria and their hosts have been tightening their relationships over millions of years. As a result, many cellular structures or genes that are necessary for free-living bacteria have been lost or transferred to the nucleus cell (Brandvain and Wade 2005), rendering mitochondria dependent on the host cell. They cannot leave the host or start to reproduce on their own. Indeed, they have to reproduce in combination with the host (with which their relationships are so ‘tight’ that Godfrey-Smith (2015) wonders whether mitochondria should be considered simple or scaffolded reproducers due to their dependence). Therefore, there are good reasons to consider a single eukaryotic cell as a collective reproducer and therefore as a unit of selection.

A less ancient but no less spectacular example of a collective reproducer can be seen in the symbiosis between the symbiotic bacteria *Buchnera* sp. and their multicellular hosts, namely aphids (Baumann 2005). Here, as opposed to the previous example, we have a multicellular host (aphid) and symbiotic bacteria. However, in this case we have as well very ‘tight’ relationships between those two units. Members of *Buchnera* sp. have lost many necessary genes in the course of evolution; it follows that they cannot perform many vital functions and thus cannot return to a free-living state. They have to live within their hosts. This is beneficial for aphids, because these microorganisms provide necessary nutrients lacking in their diet (essential amino acids). Thus, to ensure the presence of these beneficial microbes in every succeeding generation, aphids transfer them to their offspring via special propagules. Here, we have another good example of collective reproduction, since these two units reproduce together, and so we are justified in arguing that they constitute a unit of selection.

## Can a holobiont act as a unit of co-operation?

In the last section we showed that host and its symbiotic microorganisms can sometimes act as a unit of selection, because their interactions are so well integrated that they function as collective reproducers. However, such interactions represent the exception rather than the rule, because the majority of microbes within a given host are not sufficiently integrated into that host to enable it to be considered a collective reproducer. This raises an obvious question: what is to be done with the concept of the holobiont? One idea is to argue that a holobiont does not constitute a meaningful unit from the perspective of evolution; however, it may be a genuine unit from the perspective of other fields of scientific investigation, such as immunity (see Pradeu 2010, 2016). This is quite possible. Nevertheless, the concept of the holobiont is still used in debates rooted in evolutionary considerations such as speciation (as mentioned at the beginning of this paper), and thus it would be useful to have an evolutionary interpretation. We believe that, provided we accept some constraints, such an interpretation can be developed. Firstly, let us introduce some examples to show that microbes that do not reproduce along with the host may nevertheless play many crucial roles.

The most radical example of interactions not based on co-reproduction between a host and symbiotic microbes is the case of vestimentiferan tubeworms such as *Riftia pachyptila* and their symbiotic bacteria (see Gibson et al. 2010; Klose et al. 2015). These worms live in the vicinity of deep-sea hydrothermal vents which emanate the sulphide-rich fluids from which they derive their primary nutrition. However, being incapable of metabolising these fluids on their own, the worms need special symbiotic bacteria with this capability. Two things which make this symbiotic system noteworthy can be pointed out here. First, so greatly do these worms depend on the energy provided by microorganisms that during metamorphosis they lose their functioning digestive tracts altogether (Gibson et al. 2010). One might think that, given this scenario, these microbes must be passed from generation to generation in special propagules, as in the aphid–*Buchnera* symbiosis, to assure that worms of the next generation will carry them within their bodies. However, another interesting thing about this symbiosis is that these microbes are acquired by worms in every succeeding generation from the environment (Klose et al. 2015). Therefore, this symbiotic system is a good example of a high level of functional integration between host and microbes, one which is not, however, assisted by collective reproduction.

Another well-known example of symbiotic interactions is mycorrhizas, in which a fungus colonises the host plant’s roots, either intracellularly, as in arbuscular mycorrhizal fungi, or extracellularly, as in ectomycorrhizal fungi. In the light of recent research, it seems that the presence of mycorrhizas is normal among species of land plants (Wang and Qiu 2006). These interactions appear to be quite beneficial for both participants: the plant transfers carbohydrates (produced during photosynthesis) to the fungus, which then feeds the plant with water and mineral nutrients taken from the soil (Harrison 2005). Therefore, in these symbiotic systems, metabolic interactions are quite ‘tight’ and interconnected. Accordingly, one might expect that evolution has fused the reproduction process of these partners to assure that these metabolic interactions will be passed on to every succeeding generation. However, just as in the example above, the reproduction process is not aligned; these associations are established de novo in every successive generation.

The presence of important symbiotic microorganisms is not limited to deep-sea worms or to plants. All animals, such as ourselves, cats, mice, cows, harbour enormous numbers of various symbiotic microorganisms which play many crucial roles. For example, studies show that the number of microbial cells within a human body is equal to the number of human cells (Sender et al. 2016). These microbial cells are, moreover, very diverse, belonging to many different species (Ley et al. 2006) and playing many crucial roles in human biology; for instance, they are engaged in the functionality of our immune system (Hooper et al. 2012) and digestive processes (Ley et al. 2006). There is also some evidence suggesting that they may influence human behaviour (Vuong et al. 2017). Thus, it is not surprising that some scholars suggest that microorganisms are changing our understanding of what it means to be human (Rees et al. 2018). However, despite their significance for our species, we have not evolved such a high level of interdependence with them that we reproduce together as a whole, as in the case of, e.g. the aphid–*Buchnera* symbiosis, because aspects such as diet (Singh et al. 2017) or ageing (Yatsunenko et al. 2012) may change the composition of our microbiota, suggesting that we are independently reproducing units. Of course, as research continues, it may turn out that some of the microbes that inhabit our gut, for example, are incorporated in our body to the extent that we reproduce together; however, this is definitely not true of a majority of symbiotic microbes (Ley et al. 2006; Singh et al. 2017; Yatsunenko et al. 2012).

All of these examples demonstrate two things: (1) not all symbiotic microorganisms are integrated into the bodies of macrobes to the extent that they reproduce as wholes; (2) they may nevertheless play many vital roles. This raises the question: how can we conceptualise these kinds of interactions from a Darwinian perspective?

Queller and Strasmann (2009, 2010, 2016) have provided a framework for these considerations by revisiting the idea of the *organism* (which we will call a unit of co-operation here), as well as the question of relative degrees of co-operation and conflict among its elements. Thus, in their view, an organism, from an evolutionary perspective, is a functional system built of elements that co-operate to sustain its stability and functionality. Accordingly, the greater the degree of co-operation (and the lesser the degree of conflict) among elements, the higher the organismality of the unit. This way of conceptualising organisms makes a great deal of sense from an evolutionary perspective, because organisms need to deal with certain environmental obstacles in every generation. For them to do so, the elements from which they are built must function in a co-ordinated way to perform certain tasks, such as development, growth, and the digestion of a particular resource, in order to survive and reproduce. Thus, seeing an organism as a system built of elements that co-operate to maintain its structure makes sense, because evolution is all about making such systems much more co-operative in order to perform tasks ‘assigned’ by the environment.

The most interesting aspect of this conceptualisation of the organism is that it is indifferent to the mode of inheritance of the interacting elements. Therefore, since the emphasis is placed on co-operation among entities, it embraces elements that are not necessarily co-inherited over generations, such as genes inside a nuclear genome, but, as in the interactions presented above, are characterised by a high level of co-operation and a low level of conflict. Indeed, two units may have evolved a high level of interdependence, sophisticated mechanisms of communication, and even some degree of integration of their biochemical machinery, but still might reproduce independently. This might happen because, for example, participation in these kinds of interactions enhances the fitness of the partners. Thus, natural selection might favour this kind of interaction over generations because it is beneficial for the partners; however, it might not lead to alignment of their reproductive process. Therefore, the result would be a highly co-operative union of objects, a union which, however, does not reproduce as a whole. This happens fairly often, as the examples from this section demonstrate.

Now, having presented the basic theoretical assumptions of this concept, we can explain why we believe that this idea may capture the ‘evolutionary identity’ of holobionts. Firstly, this identity is less limited than the concept of being a unit of selection—a concept which requires collective reproduction, which is not present in many host–microbe interactions. Indeed, this evolutionary identity—as opposed to the idea of the Darwinian individual—does not presume that units must be co-inherited. From the perspective of this idea, a theoretical unit A and a unit B might be co-inherited, and a unit C acquired horizontally; together, they would still qualify as a valid unit of co-operation if they were characterised by a low level of conflict and high level of co-operation. Therefore, this concept captures a basic fact about evolution, namely that it can sometimes achieve the same result via different paths. In this case, a high level of integration might be achieved through aligning the process of reproduction, which ‘forces’ two units to co-operate because their fate is linked, or through evolving mechanisms to ‘find’ a partner in every succeeding generation because co-operation increases the fitness of both partners. And this is how holobionts undergo evolution, because they are basically a mix of vertically and horizontally inherited microbes.

Secondly, being a unit of co-operation is a continuous variable, in light of the various degrees of co-operation and conflict among species in nature; this is a very welcome detail, given that host–microbe interactions are characterised by various degrees of conflict and co-operation. Some microbes are important to the functionality of the host, while others demonstrate a low level of co-operation, or are even pathogenic, as medical studies teach us. In this context, we should ask not whether a particular host and its symbiotic microbes constitute an organism, but rather what degree of organismality characterises them.

In this context, it seems that the idea of the holobiont (a host and its associated microbes) represents, in fact, a unit of co-operation: a system built of co-operating units, some of which may be co-inherited along with the host (and thus act along with the host as a single Darwinian individual), while others are linked only via functional integration—which, furthermore, differs among symbiotic microorganisms. Therefore, we think that this idea captures, much better than the concept of a unit of selection, the intuitions behind the hologenome theory of evolution, as expressed by Theis et al. (2016, p. 2): ‘Microbial genomes can be stable or labile components of the hologenome and can be vertically or horizontally transmitted, and the traits that they encode are context-dependent and may result in damage, benefit, or no consequence to the holobiont’. Indeed, considering holobionts as units of co-operation enables us to retain this thinking about holobionts, because the concept of a unit of co-operation is much broader and liberal than that of a unit of selection, which is based on very rigid assumptions, including an aligned process of reproduction.

This approach has some limitations which need to be stressed. The fundamental one is that holobionts can be considered units of co-operation only if our research is focused on understanding the evolution of the host—that is, only if the focal unit of research is the host. This is because the host, due to its multicellular structure, is characterised by a very large surface area and thus is able to interact with enormous numbers of symbiotic microorganisms. Some of these interactions might be based to a greater extent on co-operation, others on conflict; thus, a holobiont might express different levels of organismality in different areas of its body (with some microbes being highly integrated, others less so). Therefore, the holobiont as a whole might not score high on the scale of organismality, yet might be considered as a unit of co-operation irrespective of this score. Unfortunately, the same cannot be said about a given associated microbe. Its surface area is much smaller, and so it cannot interact with the entire holobiont, but only with certain host cells and with certain microbes in the vicinity. This may seem to be a major disadvantage; however, if we recall that the idea of the hologenome was intended to basically capture the role of symbiotic microorganisms in the evolution of multicellular organisms (Zilber-Rosenberg and Rosenberg 2008), and that scientists still use it for that reason, then the concept of a unit of co-operation seems to serve this purpose very well.

So far, we have distinguished two kinds of entities that microbes might make up when interacting with hosts. One kind encompasses Darwinian individuals, the other units of co-operation. Thus, you, the reader, may think that a given holobiont is either an organism or a Darwinian individual. However, this is not accurate. Sometimes these two categories overlap. For instance, *Buchnera* sp. and aphids co-operate on a large scale, are characterised by a low degree of conflict, and reproduce as a single unit. Therefore such a combination constitutes both a reproducer (a unit of selection) and an organism (a unit of co-operation). Alternatively, mycorrhizal fungi engage in co-operation with plants, but do not reproduce in conjunction with them; thus, they constitute a unit of co-operation, but not a unit of selection.

These conclusions may be alarming, because the idea that a host and its associated microbes constitute a unit of selection has been used to support the statement that microbes play a role in the process of speciation (Brucker and Bordenstein 2012, 2013; Wang et al. 2015); here, we basically argue that in a majority of cases, host–microbe interactions should not be considered units of selection, but rather units of co-operation. In the next section we will show that this is not a big stumbling block for the theory of speciation, because microbes need not (but, of course, may) act with a host as a single Darwinian individual in order to cause hybrid incompatibility. Indeed, it is sufficient for them to be linked with the host in terms of functional relations only.

## Speciation and microorganisms

The current understanding of speciation is based on Bateson–Dobzhansky–Muller incompatibility (BDM), a theory developed to explain how incompatibilities between closely related species evolve to cause hybrid lethality or reduced fitness. The whole concept is based on the idea that hybrid incompatibilities are mainly the effects of interactions between products of different nuclear genes (Dobzhansky 1937; Coyne and Orr 2004; Brucker and Bordenstein 2012). These incompatibilities may result in the unsuccessful mating of genetically different individuals, resulting in no offspring or offspring with major aberrations. This occurs because the process of evolution is very dynamic and a given population may, as evolution proceeds, be divided into many geographically isolated subpopulations. Furthermore, in each of them, natural selection may favour the evolution of different traits due to ecological differences, which may lead, in turn, to genetic diversification. Thus, if two subpopulations, following a period of isolation, are somehow reunited and their members begin to mate, their genes may be found in the genome of a hybrid. However, because the subpopulations evolved independently, their genes may not interact in a very synchronised manner and thus may cause some problems capable of influencing the fitness of such a hybrid.

Therefore, the cause of hybrid incompatibility, in this classic, general view, can be found: (1) in the interaction of gene products, due to the fact that they are, for certain reasons, incompatible, and, furthermore, (2) in genes located within the nucleus cell. Therefore, the question is whether microbial genes (regardless of whether they are transmitted horizontally or vertically) can also cause hybrid incompatibility. And if so, how can we reconcile the classic view presented above with these discoveries? We will address the first question before turning to the second.

One paper relevant to our discussion is the work of Brucker and Bordenstein (2013), who studied closely related species of *Nasonia*, a genus of parasitoid wasps, and presented the idea that their hybrid incompatibilities are caused by epistatic interactions, not only between nuclear loci but also between the genes of the hosts and those of the microorganisms that live inside their guts. Specifically, following hybridisation, bacterial constituents and abundance were found to be irregular in hybrids relative to parental controls, leading to increases in mortality. This, in turn, showed that wasps develop properly only in a ‘neighbourhood’ of suitable bacterial constituents. This idea was supported by the fact that antibiotic ‘curing’ of already-present gut bacteria significantly increased hybrid survival. The researchers thus concluded that the gut microbiome and host genome represent a co-adapted ‘hologenome’ that breaks down during hybridisation, promoting hybrid lethality and assisting speciation.

Another example can be found in a paper by Wang et al. (2015), whose object of research encompassed hybrids of two subspecies of mice, namely *Mus musculus musculus* and *M. musculus domesticus.* Hybrids were caught throughout the hybrid zone of these two subspecies in Central Europe as well as generated in laboratory settings. The fundamental outcome of the research was the discovery of altered microbiomes in hybrids as compared with parents. Furthermore, genetic and immunological analysis of these hybrids revealed genetic incompatibilities, aberrant immune gene expression, and increased intestinal pathology associated with an altered community structure among hybrids. This suggests that microbiomes may be involved in the emergence of hybrid incompatibility.

Both of the papers cited above show that symbiotic microorganisms may be the agents responsible for the speciation of multicellular hosts. Of course, since science is based on empirical evidence and since we are just beginning to understanding the role of microbes in speciation, it may turn out that some of the current evidence is faulty. Nevertheless, it seems that research on microbiomics is forcing us to develop a new framework for understanding speciation, one capable of incorporating the discoveries of microbiomics. However, such ideas, emphasising the importance of symbiotic microorganisms during speciation, have not yet become well established among evolutionary biologists (Coyne and Orr 2004; Chandler and Turelli 2014). This should come as no surprise, as the results of the work of Brucker and Bordenstein (2013) or of Wang et al. (2015), at first glance, are not congruent with the orthodox approach to speciation described above, despite statements that the hologenome theory of speciation is a simple extension of BDM incompatibility: ‘[a] simple, microbial extension of the BDM model is to replace that of nuclear genes with that of microorganisms’ (Brucker and Bordenstein 2012, p. 446). We think, however, that some slight modifications of this theory would enable it to incorporate microbiological discoveries easily and expand our understanding of speciation.

BDM incompatibility is aimed at providing a good theoretical understanding of why crossed organisms originated from two independently evolving populations might fail to produce viable offspring. In this view, the cause of hybrid incompatibility is seen exclusively in non-co-operating hybrid elements (genes) inherited vertically from parents. This approach to speciation is a consequence of the Modern Synthesis paradigm; organisms were understood by contemporary scientists in the classic way, specifically, as a group of cells, the product of successive divisions of an egg cell, which interact with one another to induce developmental pathways, and which, as a whole, constitute the organism. This is one of the most popular ways of conceptualising organisms (see Pradeu 2010; Gilbert et al. 2012; Stencel and Proszewska 2017). Furthermore, this view of what constitutes an organism was maintained over the years as well by others, even by those who aimed to undermine the basic concepts of evolutionary biology, such as Dawkins (1982, p. 263): ‘The organism has the following attributes. It is either a single cell, or, if it is multicellular, its cells are close genetic kin to each other: they are descended from a single stem cell, which means that they have a more recent common ancestor with each other than with any other cells of any other organism.’

Therefore, in this view, an organism develops from a fertilised (or, in some cases, unfertilised) egg, and so all its functions must be coded by nuclear genes, because the only genetic material it gets is the nuclear material each cell inherits during mitosis. Therefore, if some of the organism’s functions fail, it is very likely because the organism has inherited genes from a genetically different parent, genes that are apparently not co-adapted to work together, and so a properly functioning multicellular being cannot emerge. Thus, in this classic understanding of speciation, a hybrid fails to function properly due to the interactions of the genes passed down from its parents during reproduction.

Unfortunately, the above understanding of organisms, which forms the basis for BDM incompatibility, is currently being questioned from many different angles, especially that of microbiomics, which shows—as we have presented throughout this paper—that many fundamental functions of organisms are performed by symbiotic microorganisms (see Pradeu 2010, 2011; Gilbert et al. 2012; Stencel and Proszewska 2017). The most radical example, presented in the previous section, is the case of vestimentiferans (tubeworms), which acquire sets of symbiotic bacteria from their environment on which they depend for their energy supply, and which, during the stage of metamorphosis, lose their functioning digestive tract altogether (Gibson et al. 2010). All of these discoveries show that entities that we know very well, such as cats, humans, etc., attain their full functionality not only by means of successive divisions from a zygote, but also through incorporating microbial cells from their surroundings.

In this context, it becomes clear that if we wish to capture the role of microbes in the speciation of macrobes, we need to switch to a more inclusive concept of the organism, one capable of incorporating discoveries about symbiotic microorganisms. Indeed, we need to use a concept of the organism that emphasises the fact that functional wholes can sometimes emerge by means of the aggregation of independently reproducing units. One good candidate is the concept of the unit of co-operation we introduced in the last section, which states that when individualising functional wholes we should look not at the way its elements are inherited, but at whether they are characterised by a high level of co-operation and a low level of conflict. In doing so, it will become clear to us that microbes may be the cause of hybrid incompatibility, because the functionality of such a whole might be reduced either by the genes of the host, as was classically understood (Dobzhansky 1937; Coyne and Orr 2004), or by those of symbiotic microbes, as recent studies show (Brucker and Bordenstein 2013; Wang et al. 2015), which fail to co-operate properly so that a functional whole cannot be established. Indeed, if the functionality of a given unit depends on the co-operation of many elements (genes of hosts and microbes), then hybrids might experience reduced fitness sometimes due to the host’s genes, sometimes to microbes’ genes. To make this point much more convincing, consider the speculative example below, which shows that reduced fitness in a hybrid is caused sometimes by the genes of symbiotic microbes and sometimes by the host’s genes.

Suppose now that we have two populations that have been evolving independently for some years. Furthermore, suppose that they have been under different selection pressure; as a result, different traits have become fixed within them. Then, somehow, members of these populations meet 1 day and hybridise. What might happen? First, a hybrid might inherit incompatible nuclear genes from its parents and its development might fail at a very early stage. Second, the genes that the hybrid inherits might co-operate very well and the hybrid might develop into a mature organism. However, such an individual might differ slightly, for instance, in the functionality of its immune system, and fail to establish relationships with beneficial microorganisms; that is, its immune system might consider certain beneficial microorganisms to be disease agents. This should come as no surprise, as it is known that the immune systems of species are adapted to interactions with specific microbes, thanks to a million years of co-evolution (Pradeu 2010, 2011). Following hybridisation, the host’s immune system might lose the ability to distinguish between beneficial and harmful microorganisms. Additionally, following hybridisation there would be no opportunity for the hybrid to acquire suitable microorganisms from its environment, and thus it might be colonised by various bacterial taxa. This scenario might occur if parents were to hybridise after leaving their standard habitat behind, which is at least theoretically possible given the geographical diversification of microbiomes (Godoy-Vitorino et al. 2012; Linnenbrink et al. 2013; Oh et al. 2010; Yatsunenko et al. 2012). As a result, irrespective of the cause, microorganisms which, for example, colonise the gut of a hybrid would lack a specific trait that, in tandem with the digestive system of the host, would have enabled them to use a specific resource. Thus, the functionality of the hybrid would be reduced as a result of interactions between nuclear and microbial gene products that failed to produce a proper metabolic network.

To sum up: to understand the role of microbes in speciation in a theoretical sense, we need not reject BDM incompatibility. Rather, we need to switch to a more inclusive understanding of functional wholes. Indeed, we need to accept that, from an evolutionary point of view, organisms—functional wholes—might emerge via an aggregation of genes passed along from parents and genes acquired from the environment. If so, then it becomes clear that microbes may be responsible for the inviability of hybrids, because the functionality of the latter might be reduced due to the lack of specific microbes that, along with the host, make up a functional, cohesive whole.

## Concluding remarks

Holobionts have recently been the objects of important empirical studies (Hosokawa et al. 2006; Ley et al. 2006; Oh et al. 2010; Yatsunenko et al. 2012; Gilbert et al. 2012; Godoy-Vitorino et al. 2012; Linnenbrink et al. 2013) which have raised many interesting theoretical questions. One important issue is to determine whether or not holobionts are genuine units from the perspective of evolution. The initial idea was to argue that holobionts were units of selection (Zilber-Rosenberg and Rosenberg 2008; Brucker and Bordenstein 2012, 2013); however, this has been questioned recently by others (Moran and Sloan 2015; Douglas and Werren 2016; Skillings 2016). Our approach is partially in line with those who question the idea that holobionts are units of selection because it places constraints on symbiotic associations, permitting the consideration of such communities as units of selection only if they function as reproducers. At the same time, our approach suggests that, by taking the host’s perspective, we can say that holobionts are units of co-operation, because hosts, due to their multicellular structure, are able to interact with enormous numbers of microbes, thus forming co-operative systems that are functionally integrated and interdependent, because this might exert a positive influence on their fitness. Furthermore, as we presented in the last section, focusing on such co-operative unions is truly important for understanding the role of microbes in the origin of new species of multicellular organisms. This is true because what actually fails during hybridisation is the functionality of the system called the organism; whether its elements are co-inherited together over generations (i.e. whether it constitutes a unit of selection) is irrelevant to the process of speciation. Thus, to incorporate microorganisms in the theory of speciation, all we need is a broader concept of the organism. The theoretical framework developed by Queller and Strasmann (2009, 2010, 2016) provides a basis for such a concept.
