# Correction to: Some theoretical insights into the hologenome theory of evolution and the role of microbes in speciation

**DOI:** 10.1007/s12064-018-0270-9

**Published:** 2018-09-21

**Authors:** Adrian Stencel, Dominika M. Wloch-Salamon

**Affiliations:** 1 Faculty of Philosophy, Jagiellonian University, ul. Gołębia 24, 31-007 Kraków, Poland; 2 Institute of Environmental Sciences, Jagiellonian University, Gronostajowa 7, 30-387 Kraków, Poland

## Correction to: Theory in Biosciences 10.1007/s12064-018-0268-3

The original version of this article unfortunately contained a mistake.

The entry Suárez, J. Symbiosis (2018). 10.1007/s13199-018-0556-1 was missing in the reference list.

The corrected reference list is given below.
